# S2-2 Characterizing the adoption of physical literacy in research, practice and policy across Europe

**DOI:** 10.1093/eurpub/ckad133.010

**Published:** 2023-09-11

**Authors:** Johannes Carl

**Affiliations:** Department of Sport Science and Sport, Friedrich-Alexander University Erlangen-Nürnberg, Germany

## Abstract

**Purpose:**

The concept of physical literacy (PL) takes a holistic perspective on physically active lifestyles by simultaneously considering physical, cognitive, and affective/psychological requirements on the personal level. In recent years, PL has undergone a dynamic development globally and Europe is no exception. Yet, there is scant knowledge about the current situation of PL in Europe. Against this background, the purpose of this study was to comprehensively assess and compare the state of PL implementation in research, policy, and practice across the continent.

**Methods:**

We assembled a panel of 34 experts across 25 European countries (snowballing principle). Drawing on a complementary mixed-methods design, the experts first prepared reviews about the current state of PL in their countries (prescribed categories: research, practice/policy). Subsequently, two researchers from different countries submitted the reviews to a four-step document analysis (material reading, data extraction, data analysis, distillation of findings). Finally, the representatives completed a quantitative survey with items reflecting the inductive themes from the document analysis to re-validate the qualitative findings.

**Results:**

The document analysis yielded ten disjunct themes (related to the overarching categories “concept”, “research”, “practice/policy”, “future/prospect”). We identified a heterogenous situation of PL in Europe, with anglophone countries tending to better adopt the concept in research, practice, and policy. Research initiatives are largely fragmented within the European countries and scholarly progress often bases on the effort of single researchers. PL plays hardly any role in health agendas on the national level and curricula often list single PL elements without explicitly mentioning PL. In summary, PL hesitantly permeates practice and policy in most countries, while the implementation state was strongly related to conceptual discussions (e.g., dominance of competing approaches), translation challenges, and country-specific traditions. Nonetheless, the country representatives anticipate an increasing popularity of PL in the future.

**Conclusions:**

Despite the heterogeneous situation in Europe, the analysis has revealed similarities among the countries, such as the presence of established yet not identical concepts. Research should strengthen the academic activities (conceptual-linguistic elaborations, empirical work) before PL may gain further access into practical and political spheres in the long term.

**Support/Funding Source:**

No specific funding.

